# Supplementary material for: Rice koji reduced body weight gain, fat accumulation, and blood glucose level in high-fat diet-induced obese mice
Source: PeerJ. 2014 Aug 26;2:e540. doi: 10.7717/peerj.540 (PMC4157231; doi:10.7717/peerj.540)
Supplement: Supplemental Information 1 — Raw data for all figures and tables. [file peerj-02-540-s001.zip › RawData/Table1_raw_data.pdf]

Japan
Food
Research
Laboratories

第 09018648001-01 号
2009年(平成21年)11月10日

分析試験成績書

依 頼 者 国立大学法人 鹿児島大学

検 体 名 蒸し米

財団法人
日本食品分析センター
東京都渋谷区元代々木町52番1号

2009年(平成21年)10月28日 当センターに提出された上記検体について分析試験した結果は次のとおりです。

分析試験結果

分析試験項目	結果	定量下限	注	方法
水分	4.1 g/100g	......		常圧加熱乾燥法
たんぱく質	7.1 g/100g	......	1	ｹﾙﾀﾞｰﾙ法
脂質	1.0 g/100g	......		酸分解法
灰分	0.3 g/100g	......		直接灰化法
炭水化物	87.5 g/100g	......	2	......
ｴﾈﾙｷﾞｰ	387 kcal/100g	......	3	......

注1. 窒素・たんぱく質換算係数:5.95
注2. 計算式:100-(水分+たんぱく質+脂質+灰分)
注3. 栄養表示基準(平成15年厚生労働省告示第176号)によるｴﾈﾙｷﾞｰ換算係数:たんぱく質,4;脂質,9;炭水化物,4

以 上

本成績書を他に掲載するときは当センターの掲載規約をお守り下さい。 財団法人 日本食品分析センター

Japan Food Research Laboratories

第 09018648002-01 号
2009年(平成21年)11月10日

分析試験成績書

依 頼 者　国立大学法人　鹿児島大学

検 体 名　黄麹

財団法人
日本食品分析センター
東京都渋谷区元代々木町52番1号

2009年(平成21年)10月28日 当センターに提出された上記検体について分析試験した結果は次のとおりです。

分析試験結果

分析試験項目	結果	定量下限	注	方法
水分	5.4 g/100g	……		常圧加熱乾燥法
たんぱく質	9.3 g/100g	……	1	ｹﾙﾀﾞｰﾙ法
脂質	2.1 g/100g	……		酸分解法
灰分	0.3 g/100g	……		直接灰化法
炭水化物	82.9 g/100g	……	2	……
ｴﾈﾙｷﾞｰ	388 kcal/100g	……	3	……

注1. 窒素・たんぱく質換算係数:5.95
注2. 計算式:100-(水分+たんぱく質+脂質+灰分)
注3. 栄養表示基準(平成15年厚生労働省告示第176号)によるｴﾈﾙｷﾞｰ換算係数:たんぱく質,4;脂質,9;炭水化物,4

以 上

Japan Food Research Laboratories

分析試験成績書

依　頼　者　国立大学法人　鹿児島大学

検　体　名　白麹

財団法人
日本食品分析センター
東京都渋谷区元代々木町52番1号

2009年(平成21年)10月28日 当センターに提出された上記検体について分析試験した結果は次のとおりです。

分析試験結果

分析試験項目	結果	定量下限	注	方法
水分	8.5 g/100g	……		常圧加熱乾燥法
たんぱく質	9.2 g/100g	……	1	ｹﾙﾀﾞｰﾙ法
脂質	1.5 g/100g	……		酸分解法
灰分	0.3 g/100g	……		直接灰化法
炭水化物	80.5 g/100g	……	2	……
ｴﾈﾙｷﾞｰ	372 kcal/100g	……	3	……

注1. 窒素･たんぱく質換算係数:5.95
注2. 計算式:100-(水分+たんぱく質+脂質+灰分)
注3. 栄養表示基準(平成15年厚生労働省告示第176号)によるｴﾈﾙｷﾞｰ換算係数:たんぱく質,4;脂質,9;炭水化物,4

以　上

本成績書を他に掲載するときは当センターの掲載規約をお守り下さい。 財団法人 日本食品分析センター　RCA0201-01

Japan Food Research Laboratories

第 09018648004-01 号
2009年(平成21年)11月10日

分析試験成績書

依　頼　者　国立大学法人　鹿児島大学

検　体　名　黒麹

財団法人
日本食品分析センター
東京都渋谷区元代々木町52番1号

2009年(平成21年)10月28日 当センターに提出された上記検体について分析試験した結果は次のとおりです。

分析試験結果

分析試験項目	結果	定量下限	注	方法
水分	5.9 g/100g	……		常圧加熱乾燥法
たんぱく質	9.0 g/100g	……	1	ｹﾙﾀﾞｰﾙ法
脂質	1.7 g/100g	……		酸分解法
灰分	0.3 g/100g	……		直接灰化法
炭水化物	83.1 g/100g	……	2	……
ｴﾈﾙｷﾞｰ	384 kcal/100g	……	3	……

注1. 窒素・たんぱく質換算係数:5.95
注2. 計算式:100-(水分+たんぱく質+脂質+灰分)
注3. 栄養表示基準(平成15年厚生労働省告示第176号)によるｴﾈﾙｷﾞｰ換算係数:たんぱく質,4;脂質,9;炭水化物,4

以　上

本成績書を他に掲載するときは当センターの掲載規約をお守り下さい。 財団法人 日本食品分析センター

Japan Food Research Laboratories

第 09018648005-01 号
2009年(平成21年)11月10日

分析試験成績書

依　頼　者　国立大学法人　鹿児島大学

検　体　名　紅麹

財団法人
日本食品分析センター
東京都渋谷区元代々木町52番1号

2009年(平成21年)10月28日 当センターに提出された上記検体について分析試験した結果は次のとおりです。

分析試験結果

分析試験項目	結果	定量下限	注	方法
水分	4.9 g/100g	……		常圧加熱乾燥法
たんぱく質	9.6 g/100g	……	1	ｹﾙﾀﾞｰﾙ法
脂質	1.8 g/100g	……		酸分解法
灰分	0.3 g/100g	……		直接灰化法
炭水化物	83.4 g/100g	……	2	……
ｴﾈﾙｷﾞｰ	388 kcal/100g	……	3	……

注1. 窒素・たんぱく質換算係数:5.95
注2. 計算式:100-(水分+たんぱく質+脂質+灰分)
注3. 栄養表示基準(平成15年厚生労働省告示第176号)によるｴﾈﾙｷﾞｰ換算係数:たんぱく質,4;脂質,9;炭水化物,4

以　上

本成績書を他に掲載するときは当センターの掲載規約をお守り下さい。 財団法人 日本食品分析センター

Japan Food Research Laboratories

第 09018648006-01 号
2009年(平成21年)11月10日

分析試験成績書

依 頼 者　国立大学法人　鹿児島大学

検 体 名　黄麹-液化

財団法人
日本食品分析センター
東京都渋谷区元代々木町52番1号

2009年(平成21年)10月28日 当センターに提出された上記検体について分析試験した結果は次のとおりです。

分析試験結果

分析試験項目	結果	定量下限	注	方法
水分	49.3 g/100g	……		減圧加熱乾燥法
たんぱく質	4.6 g/100g	……	1	ｹﾙﾀﾞｰﾙ法
脂質	0.8 g/100g	……		酸分解法
灰分	0.2 g/100g	……		直接灰化法
炭水化物	45.1 g/100g	……	2	……
ｴﾈﾙｷﾞｰ	206 kcal/100g	……	3	……

注1. 窒素・たんぱく質換算係数:5.95
注2. 計算式:100-(水分+たんぱく質+脂質+灰分)
注3. 栄養表示基準(平成15年厚生労働省告示第176号)によるｴﾈﾙｷﾞｰ換算係数:たんぱく質,4;脂質,9;炭水化物,4

以　上

本成績書を他に掲載するときは当センターの掲載規約をお守り下さい。 財団法人 日本食品分析センター

分析試験成績書

依　頼　者　国立大学法人　鹿児島大学

検　体　名　白麹-液化

財団法人
日本食品分析センター
東京都渋谷区元代々木町52番1号

2009年(平成21年)10月28日 当センターに提出された上記検体について分析試験した結果は次のとおりです。

分析試験結果

分析試験項目	結果	定量下限	注	方法
水分	49.8 g/100g	……		減圧加熱乾燥法
たんぱく質	4.7 g/100g	……	1	ｹﾙﾀﾞｰﾙ法
脂質	0.6 g/100g	……		酸分解法
灰分	0.2 g/100g	……		直接灰化法
炭水化物	44.7 g/100g	……	2	……
ｴﾈﾙｷﾞｰ	203 kcal/100g	……	3	……

注1. 窒素・たんぱく質換算係数:5.95
注2. 計算式:100-(水分+たんぱく質+脂質+灰分)
注3. 栄養表示基準(平成15年厚生労働省告示第176号)によるｴﾈﾙｷﾞｰ換算係数:たんぱく質,4;脂質,9;炭水化物,4

以　上

本成績書を他に掲載するときは当センターの掲載規約をお守り下さい。 財団法人 日本食品分析センター

Japan Food Research Laboratories

第 09018648008-01 号
2009年(平成21年)11月10日

分析試験成績書

依 頼 者　国立大学法人　鹿児島大学

検 体 名　黒麹-液化

財団法人
日本食品分析センター
東京都渋谷区元代々木町52番1号

2009年(平成21年)10月28日 当センターに提出された上記検体について分析試験した結果は次のとおりです。

分析試験結果

分析試験項目	結果	定量下限	注	方法
水分	48.7 g/100g	……		減圧加熱乾燥法
たんぱく質	4.6 g/100g	……	1	ｹﾙﾀﾞｰﾙ法
脂質	0.8 g/100g	……		酸分解法
灰分	0.2 g/100g	……		直接灰化法
炭水化物	45.7 g/100g	……	2	……
ｴﾈﾙｷﾞｰ	208 kcal/100g	……	3	……

注1.　窒素・たんぱく質換算係数:5.95

注2.　計算式:100-(水分+たんぱく質+脂質+灰分)

注3.　栄養表示基準(平成15年厚生労働省告示第176号)によるｴﾈﾙｷﾞｰ換算係数:たんぱく質,4;脂質,9;炭水化物,4

以　上

本成績書を他に掲載するときは当センターの掲載規約をお守り下さい。　財団法人 日本食品分析センター　RCA0201-01

Japan
Food
Research
Laboratories

第 09018648009-01 号
2009年(平成21年)11月10日

分析試験成績書

依　頼　者　国立大学法人　鹿児島大学

検　体　名　紅麹-液化

財団法人
日本食品分析センター
東京都渋谷区元代々木町52番1号

2009年(平成21年)10月28日 当センターに提出された上記検体について分析試験した結果は次のとおりです。

分析試験結果

分析試験項目	結果	定量下限	注	方法
水分	48.7 g/100g	……		減圧加熱乾燥法
たんぱく質	4.8 g/100g	……	1	ｹﾙﾀﾞｰﾙ法
脂質	0.7 g/100g	……		酸分解法
灰分	0.2 g/100g	……		直接灰化法
炭水化物	45.6 g/100g	……	2	……
ｴﾈﾙｷﾞｰ	208 kcal/100g	……	3	……

注1. 窒素・たんぱく質換算係数:5.95
注2. 計算式:100-(水分+たんぱく質+脂質+灰分)
注3. 栄養表示基準(平成15年厚生労働省告示第176号)によるｴﾈﾙｷﾞｰ換算係数:たんぱく質,4;脂質,9;炭水化物,4

以　上

本成績書を他に掲載するときは当センターの掲載規約をお守り下さい。 財団法人 日本食品分析センター
